# Microsatellite Markers Reveal Strong Genetic Structure in the Endemic Chilean Dolphin

**DOI:** 10.1371/journal.pone.0123956

**Published:** 2015-04-21

**Authors:** María José Pérez-Alvarez, Carlos Olavarría, Rodrigo Moraga, C. Scott Baker, Rebecca M. Hamner, Elie Poulin

**Affiliations:** 1 Instituto de Ecología y Biodiversidad (IEB), Facultad de Ciencias, Universidad de Chile, Ñuñoa, Santiago, Chile; 2 Centro de Investigación Eutropia, Santiago, Chile; 3 Fundación CEQUA, Punta Arenas, Chile; 4 Marine Mammal Institute and Department of Fisheries and Wildlife, Oregon State University, Hatfield Marine Science Center, Newport, Oregon, United States of America; National Cheng-Kung University, TAIWAN

## Abstract

Understanding genetic differentiation and speciation processes in marine species with high dispersal capabilities is challenging. The Chilean dolphin, *Cephalorhynchus eutropia*, is the only endemic cetacean of Chile and is found in two different coastal habitats: a northern habitat with exposed coastlines, bays and estuaries from Valparaíso (33°02′S) to Chiloé (42°00′S), and a southern habitat with highly fragmented inshore coastline, channels and fjords between Chiloé and Navarino Island (55°14′S). With the aim of evaluating the potential existence of conservation units for this species, we analyzed the genetic diversity and population structure of the Chilean dolphin along its entire range. We genotyped 21 dinucleotide microsatellites for 53 skin samples collected between 1998 and 2012 (swab: n = 8, biopsy: n = 38, entanglement n = 7). Bayesian clustering and spatial model analyses identified two genetically distinct populations corresponding to the northern and southern habitats. Genetic diversity levels were similar in the two populations (He: 0.42 v/s 0.45 for southern and northern populations, respectively), while effective size population was higher in the southern area (Ne: 101 v/s 39). Genetic differentiation between these two populations was high and significant (F_ST_ = 0.15 and R_ST_ = 0.19), indicating little or no current gene flow. Because of the absence of evident geographical barriers between the northern and southern populations, we propose that genetic differentiation may reflect ecological adaptation to the different habitat conditions and resource uses. Therefore, the two genetic populations of this endemic and Near Threatened species should be considered as different conservation units with independent management strategies.

## Introduction

The marine environment provides an opportunity to examine speciation processes and population structure in species with high dispersal capabilities, generally in the absence of obvious barriers to gene flow [1,2]. In cetacean populations, a lack of geographical barriers does not necessarily result in large panmictic populations [3,4], and a genetic population pattern is difficult to interpret from a simple assessment of geography [5]. Instead, a combination of complex behavioral specialization for local resources, social structure and in some cases historical environmental changes have been associated with the population structure pattern of these species [4–8].

Populations of small cetaceans found in coastal habitats often show a population structure pattern related to geographic distance or environmental characteristics [9]. For example, for bottlenose dolphins, *Tursiops truncatus*, distributed across the contiguous range from the Black Sea to eastern North Atlantic Scotland, a population structure that coincided with transitions between habitat regions has been reported. These regions can be characterized by ocean floor topography, and oceanographic features such as surface salinity, productivity and temperature [10]. Similar patterns of population differentiation where genetic structure is shaped by oceanographic and environmental characteristic has been described for harbor porpoises, *Phocoena phocoena*, in the south-eastern North Atlantic [2] and in a medium to large spatial scale, for short-beaked common dolphin, *Delphinus delphis* from the Atlantic, Pacific and Indian Oceans [11].

A reliable understanding of the historic/contemporary genetic variation, demographic partitions throughout the geographical range and of the distribution of genetic diversity is crucial to devise effective and sustainable management plans for species [12, 13]. Genetic conservation studies provide information about the genetic structuring of the species and operationally, can be used to delineate conservation units, which include evolutionary significant units (ESUs) and management units (MUs) [14].

Dolphins of the genus *Cephalorhynchus* are distributed in inshore waters of the Southern Hemisphere [15] and appear to be depth limited in habitat preference [16,17]. A common ancestor from South African waters has been suggested for *Cephalorhynchus* dolphins based on mtDNA analysis. Following the West Wind Drift, these dolphins would have colonized New Zealand and then South America [15]. Four species are currently recognized for the *Cephalorhynchus* genus (*C*. *heaviside*, *C*. *hectori*, *C*. *commersonii* and *C*. *eutropia*). Studies on population genetic structuring have been conducted on three of these species. For Hector’s dolphin, endemic to New Zealand, two subspecies have been recognized based on genetic and morphological evidences: *C*. *hectori maui* for the North Island population and *C*. *hectori hectori* for the South Island populations [18,19]. Significant differentiation among several populations around the South Island has been also identified by genetic analysis of a large sample of mtDNA and an extensive survey of microsatellites [19–22]. Based on this information, different MUs with specific conservation strategies have been implemented for each *C*. *hectori* subspecies independently [23]. In Commerson´s dolphin two subspecies have also been described: *C*. *c*. *commersonii*, found in shallow waters of the southeastern coast of South America, including the central and eastern Strait of Magellan and waters around the Falkland Islands/Islas Malvinas [24,25] and *C*. *c*. *kerguelenensis*, distributed around the sub-Antarctic Kerguelen Islands in the southern Indian Ocean [26,27]. Genetic differentiation of mtDNA in *C*. *c*. *commersonii* showed differences among areas within Tierra del Fuego [28] and along the southern Argentina coastline [29]. Unlike Hector's and Commerson's dolphins, the Heaviside's dolphin, *Cephalorhynchus heavisidii*, endemic to coastal waters of the western coast of South Africa and Namibia, shows no evidence of population structure based on mtDNA analyses along almost 1000 nmi of the South African/Namibian coast [30]. Finally, for the Chilean dolphin, *Cephalorhynchus eutropia*, a genetic study of mtDNA extracted from teeth of museum specimens focused on resolving uncertainty in species identification [31]; however, no genetic diversity or population structure study have been undertaken for this species.

The Chilean dolphin, is the only endemic cetacean species of Chile [32], although recently a few individuals have been reported in the Argentine coast [33]. It inhabits two different but contiguous coastal habitats: a northern habitat with exposed coastlines, bays and estuaries from Valparaíso (33°02′S) to Chiloé (42°00′S), and a southern habitat with highly fragmented inshore coastline, channels and fjords between Chiloé and Navarino Island (55°14′S) [34]. Most of the sightings of the Chilean dolphin have been recorded near the shore in shallow waters, and occasionally in estuaries and rivers [24,35,36]. An estuarine-influenced habitat preference has been suggested for the species in the central coast of Chile, where aggregations of tens to hundreds dolphins have been reported in the open coast of Valdivia [37] and at the mouth of the Maule River [38]. In the southern distribution area, sightings of the species in the channels and fjords are scarce. In general, group sizes are small, ranging from 2–10 individuals; however, overall abundance has not been estimated [39,40].

Initially, the Chilean dolphin was classified as Data Deficient (DD) due to the insufficient information available to conduct a reliable assessment of its conservation status, in particular because of the lack of demographic information on population trends [41]. However, recently, the classification of this species has been changed to Near Threatened [42], considering that human impacts may have severely reduced their distribution and abundance [39]. The main reported impacts for *C*. *eutropia* during the last decade were its use as bait in southern king crab fisheries in southern Chile [43,44,45] and to a lesser extent for human consumption [46]. Currently, the threats are mainly related to incidental entanglement in artisanal fisheries as well as habitat degradation associated with intense mariculture activities [39].

Considering that the identification of conservation and management units based on genetic evidence provide essential information for the development of effective and efficient conservation practices [47] this study assess the genetic diversity and population structure of the Chilean dolphin along its distribution range. Therefore, we analyzed microsatellites to evaluate the potential existence of a single conservation unit for this endemic and Near Threatened species based on its continuous distribution along the Chilean coast.

## Materials and Methods

**Fig 1:**
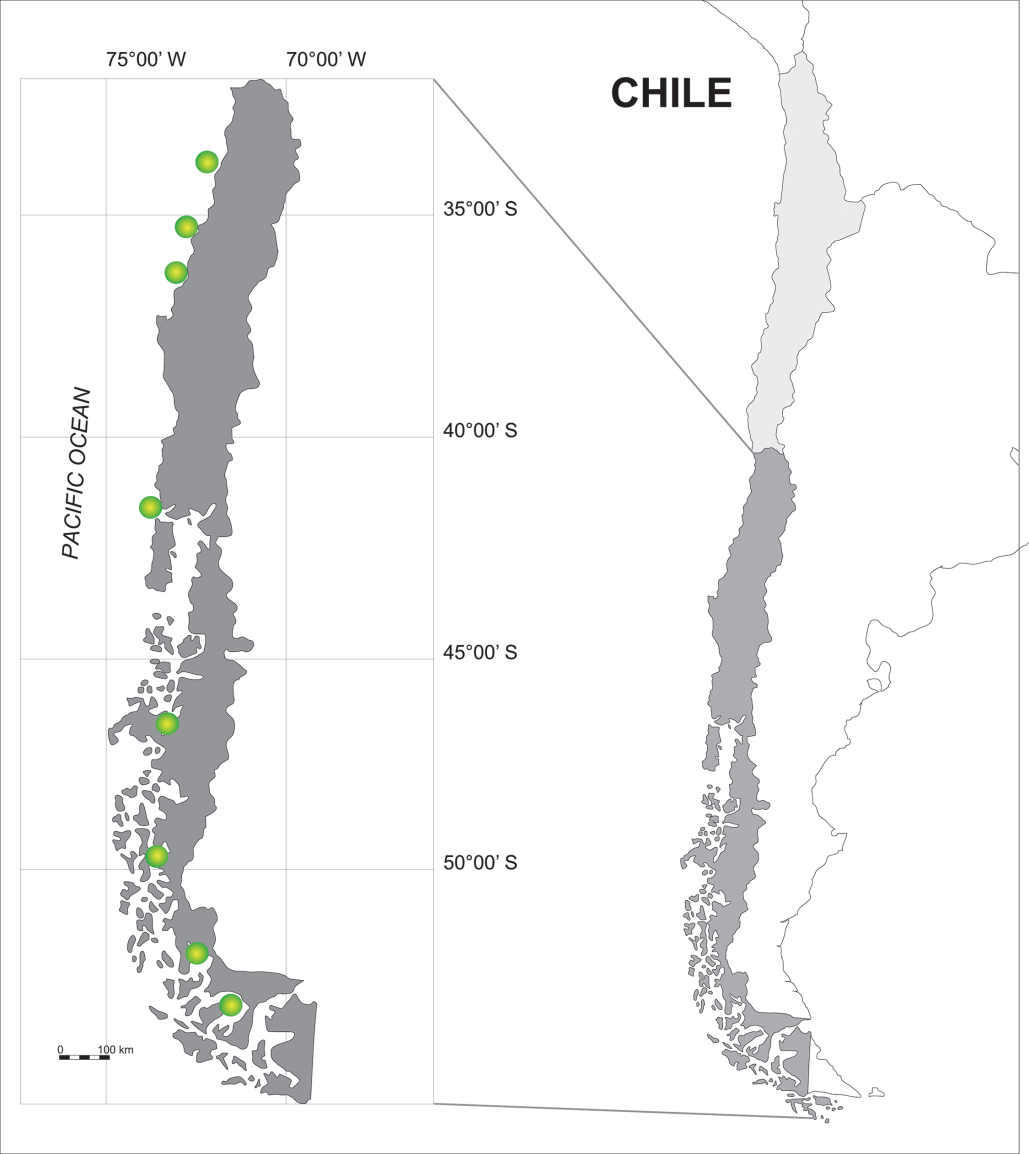
Locations where Chilean dolphins *Cephalorhynchus eutropia* were sampled. Circles correspond from north to south to: San Antonio, Constitución, Concepción, Maullín, Aysén, Bernardo O´Higgings, Puerto Natales and Punta Arenas.

Between 1998 and 2012, a total of 66 tissue samples were collected from Chilean dolphins at 8 localities along the Chilean coast ranging from 35°20′S to 52° 40'S (Fig 1). Skin samples were obtained from free-swimming adult dolphins by skin swabbing (n = 10) [48] and biopsy darting (n = 41) [49]. Additionally skin samples from different parts of the body of freshly entangled animals were also collected (n = 15, S1 Table). Samples were stored in 90% ethanol and DNA extraction followed the salt extraction method [50]. The skulls from entangled individuals were reviewed to confirm the species identification, based on skull diagnostic characteristics [51]. Samples were collected under permit from the Chilean Under Fisheries RES 665/2009, RES 67/2010 and RES 334/2012 and approved by the Bioethical Committee at the Universidad de Chile.

### Genetic sex identification

The sex of each individual was identified using two sets of oligonucleotide primers simultaneously [52], which amplify a fragment from the ZFX/ZFY genes [53] and another fragment from the SRY [52]. Sex identification was completed 2–3 times per individual and DNA from an individual of known sex was amplified as positive control.

### Genotyping

Samples were genotyped at 22 dinucleotide microsatellite loci: EV1, EV14, EV37, EV94, EV104 [54]; KWM12 [55]; MK5, MK6 [56]; PPH110, PPH130, PPH137, PPH142 [57]; GT023, GT211, GT575 [58]; TtruGT51, TtruGT142 [59]; Sgui02, Sgui03, Sgui06, Sgui17 [60] and TexVet5 [61].

Amplification reactions contained 25–50 ng DNA, PCR buffer, 5 mM MgCl2, 0.4 μM of each primer, 0.2 mM deoxynucleoside triphosphate (dNTP) and 0.5 units Platinum *Taq* DNA polymerase (Invitrogen Life Technologies). The thermal cycler profile consisted of a preliminary denaturing period of 3 min. at 94°C followed by 30 cycles of denaturation for 30 s at 94°C, primer annealing for 30 s with variable temperature (45°C to 55°C) depending of primer used, polymerase extension for 30 s at 72°C. A final extension period for 10 min. at 72°C was included. Following amplification and genotyping, allele peaks were visually verified using *Gene Marker* (Softgenetics). To minimize genotyping error each amplification and size run included a negative control to detect contamination and at least two internal control samples to ensure comparable allele sizing across all runs. Additionally three blind replicates were independently run for 10 loci.

### Microsatellite analyses

The dataset was initially checked for genotyping or scoring errors caused by null alleles, stuttering and short allele dominance using *Microchecker v*. *2*.*2*.*3* [62]. Re-sampled individuals were identified by comparing genotypes in *Cervus v*. *3*.*0* [63] and *Pedigree v*. *2*.*0* [64]. To avoid false exclusion, initial matching allowed for up to five mismatching loci, re-examining visually all the cases. Sex was subsequently compared to support our confidence in correctly identifying re-samples.

To determine spatial population boundaries we used *Geneland* [65], a Bayesian-based program that uses genotypes and spatial coordinates of individuals to cluster them into populations at approximately Hardy-Weingberg equilibrium, considering linkage equilibrium between loci. An allele frequency uncorrelated model was set, with 1,000,000 MCMC iterations and thinning of 100. Population structure was also evaluated through the Bayesian clustering method implemented in *Structure v*. *2*.*3*.*1* [66]. The admixture model with correlated allele frequencies was used without specifying sampling locations. The model was run with the likely number of clusters (K) set to values from 1 to 9 using a burn-in period of 100,000 iterations followed by 500,000 Markov chain Monte Carlo (MCMC) iterations. Five independent runs were conducted for each value of K to check for convergence of results. The number of clusters or populations (K) was inferred from the posterior probability distribution Pr (K/X) calculated from the posterior probability of the data Log Pr (X/K). Additionally, in order to corroborate and visualize the number of populations identified, the Evanno method was implemented in the *Structure Harvester* program [67,68]. Assignment of individuals to their putative populations identified by *Geneland* and *Structure* programs was verified by the Bayesian method of Rannala and Mountain [69] with an alpha of 0.01 and 10,000 repetitions of the Paetkau *et al*. [70] MCMC re-sampling algorithm implemented in *Geneclass v*. *2*.*0* [71]. Fisher Exact test conducted in *Genepop* [72] was performed to evaluate the population structure between populations identified through the Bayesian clustering methods, considering all the individuals and also males and females separately in order to test sex-biased dispersal. Level of genetic differentiation was estimated by computing Weir & Cockerham’ F_ST_ [73] in *Genetix* [74], while the R_ST_ value was calculated by *FSTAT* Software [75]. The statistical significance of genetic differentiation indices was estimated using permutation tests with 10,000 iterations.

Contemporary gene flow between these populations was estimated by Bayesian inference of recent migration rate using multilocus genotype implemented in *Bayesass* Software [76]. The delta values for migration rate were kept at the program default (0.15). The burn-in period was set at 1,0000,000, and the iterations were set to 3,000,000. The sampling frequency was set to 2,000. Analysis using optimum model parameters showed variability with different initial seed values.

Deviation from Hardy-Weinberg equilibrium and linkage disequilibrium were analyzed for each population identified through the Bayesian clustering methods. We performed a permutation test using 10,000 iterations using *Genetix* [74]. Genetic diversity was estimated by calculating allelic richness (AR) per population by *FSTAT* Software [75]; expected (He) and observed (Ho) heterozygosity per locus and per population using *Arlequin v*. *3*.*5* [77]. Effective population size (Ne) was estimated with LDNE [78] which implements a method for estimating Ne based on random linkage disequilibrium (LD) that arises due to random genetic drift in a finite population [79].

## Results

All entangled individuals were correctly assigned to *Cephalorynchus eutropia* based on the diagnostic characteristics of the species as left premaxillary bone length, exposition of the frontal bone, size of the optic foramen and rostrum length [51].

From the 66 dolphin samples, 55 samples were successfully genotyped for all 22 loci. Unsuccessful amplification was mainly associated with skin swabbing samples. Additionally, two samples were excluded from further analyses based on having identical genotypes and sex. Of the 53 individuals, 32 were males and 21 were females. Replicates for sex identification and genotyping did not shown any mismatches. The overall sex ratio did not differ from the expected 1:1 (exact Two-Tail binomial test, P = 0.17). Do not evidence for genotyping, scoring errors or null alleles was detected using *Microchecker*, except for locus Sgui02, which exhibited null alleles, and was eliminated for further analyses.

### Identification of population units

**Fig 2:**
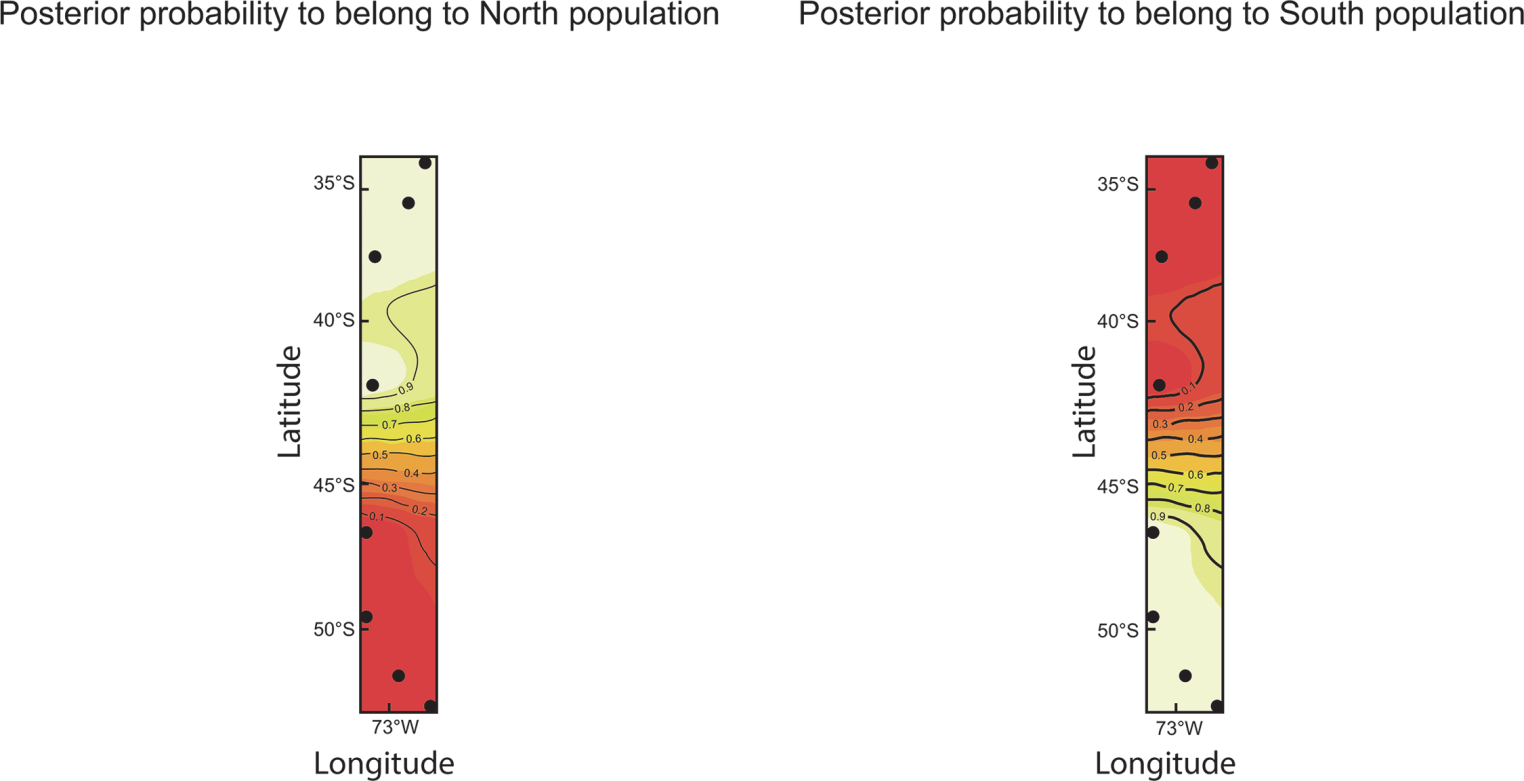
Posterior probabilities of population membership and genetic discontinuities from the spatial model in GENELAND for the Chilean dolphin. Contour lines indicate the spatial position of genetic discontinuities and lighter colors indicate higher probabilities of population membership. Two genetic clusters were identified. Left: North area, right: South area

**Fig 3:**
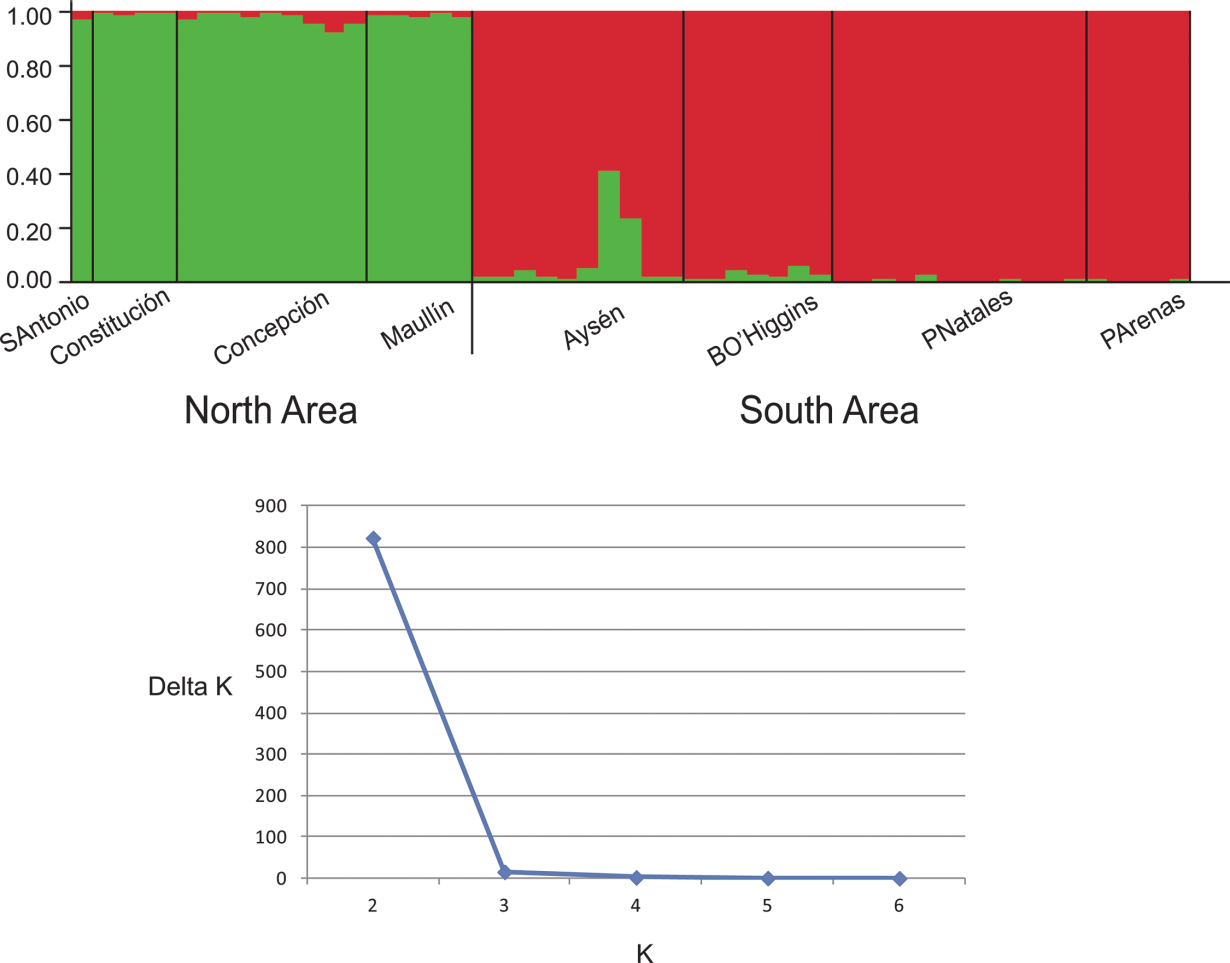
Bayesian clustering from STRUCTURE for the Chilean dolphin (above) and Evanno method in STRUCTURE HARVESTER (below). The most likely number of genetic clusters in the data set was identified as two. Each individual is represented by a vertical column partitioned into colored segments, with the length proportional to the individual´s estimated membership coefficient for the two groups: North and South areas.

The spatial model of *Geneland* identified two clusters, a ‘North area’ population including the four sampled locations from San Antonio to Maullín, and a ‘South area’ population including the four sampled locations from Aysén to Punta Arenas, with a boundary between Maullín (41,6°S) and Aysén (46,6°S) (Fig 2). The individuals sampled from the North area had a very high probability of belonging to cluster 1, and those from the South area of belonging to cluster 2 (Fig 2). Results from the Bayesian clustering approach implemented in *Structure* and the Evanno method in *Structure Harvester* also suggested two as the most likely number of groups (Fig 3). These two groups correspond to the same geographical units identified by *Geneland*. When analyzed separately, ‘North area’ and ‘South area’ populations did not show any evidence for further structuring. Genetic differentiation between these northern and southern populations was highly significant (Exact test p<0.0001; F_ST_ = 0.15 p<0.0001; R_ST_ = 0.19, p<0.001) and strong population differentiation was also observed for both males (Fisher exact test p<0.0001; F_ST_ = 0.13 P<0.002) and females (Fisher exact test p<0.0001; F_ST_ = 0.11 p<0.003)

No first generation migrants between regions were detected by *Structure* (Fig 3) or *Geneclass* (100% of the individuals were correctly assigned to their putative population; Quality Index 97.45%). However, two individuals sampled by skin swabbing in Aysén could be considered as second or third generation migrants (Fig 3). Contemporary migration rate per generation was very low in both directions (m = 0.014 ±0.013 from North to South and m = 0.020±0.018 for South to North).

No significant deviations from Hardy–Weinberg equilibrium across populations were detected (North area: F_IS_ = -0.019, P = 0.65; South area: F_IS_ = 0.041, P = 0.12) and no linkage disequilibrium was found over 210 comparisons between loci per population after Bonferroni correction. No difference in heterozygosity values was detected between the North area and the South area (He: 0.45 v/s 0.42 respectively, X^2^ = 0.18, df = 1, P = 0.66). Seven out of 21 loci had greater allelic richness in the North area while 14 loci exhibited greater allelic richness in the South area (S2 Table). Thirteen versus 29 private alleles were found in the North and South area respectively. The long-term effective population size (N_e_) showed lower values in the North (39 individuals) area than in the South (101 individuals).

## Discussion

### Population subdivision

Two genetic populations of Chilean dolphin were identified by 21 microsatellite loci; the North area population from San Antonio to Maullín and the South area population from Aysén to Punta Arenas (see Fig 1). The differentiation index values for these two population (F_ST_ = 0.15 and R_ST_ = 0.19) were generally higher than those reported among populations of other dolphin species: at a large geographic scale, F_ST_ = 0.034 for a comparison between bottlenose dolphins of coastal waters of the Gulf of Mexico and those inhabiting the waters of the western North Atlantic [4] and F_ST_ = 0.02–0.09 for a worldwide phylogeography of common dolphins, *Delphinus sp*.[80]. At smaller geographic scale, F_ST_ = 0.05 was reported for shorth-beaked common dolphins along the East Australian Coast, [8]; F_ST_ = 0.05 for inshore Indo-Pacific bottlenose dolphin, *Tursiops aduncus*, in Moreton Bay, Austalia, [81]. Differentiation values similar to those found between the Chilean dolphin populations in this study are typically reported between geographically distant populations or those elevated to subspecies. For example, harbor porpoises, have high F_ST_ = 0.14–0.314 between the Black Sea and eastern Atlantic, but much lower F_ST_ = 0.001–0.09 among continuous areas in the eastern North Atlantic [2]. The New Zealand endemic Hector’s dolphin subspecies also provides an interesting contrast to the congener Chilean dolphin.

The genetic differentiation reported between *C*. *hectori hectori* and *C*. *hectori maui* (F_ST_ = 0.167; [16]) is similar to that of the North area and South area populations of the Chilean dolphin (F_ST_ = 0.15, R_ST_ = 0.19). However, in contrast to the Chilean dolphin, these populations are discontinuous, separated by the Cook Strait, which is likely acting as a barrier to dispersal and gene flow between North and South Islands [82,83,84]. On a smaller geographic scale, the four populations of Hector’s dolphins around the South Island (separated by at least 100 km) show substantially lower genetic differentiation (F_ST_ = 0.039–0.071, p<0.005; [19]) than the two contiguous populations of Chilean dolphins. Factors such as avoidance of deep water [82] associated with a limited home range (estimated around 31 to 33 km, [82,83,85] have been proposed as contributing to genetic structure of the Hector´s dolphin at such a limited geographic scale.

### Reduced gene flow of Chilean dolphins across a main biogeographic boundary

The pattern of genetic structure observed in the present study appears to coincide with two major marine spatial biogeographic units: (1) an area located from 30°S to 42°S (Intermediate Zone or Central /southern region) characterized by an open and exposed coast with the presence of river runoff [86] where the Chilean dolphin shows an estuarine habitat preference [37,38,87,88] and (2) a southern area from 42°S to 56°S called the Magellan Province [89] or the Austral Fjords Region [90]. This southern area is a protected area of fjords and channels characterized by water in the inlets which originates from Subantarctic water and the melting of resident glaciers [90]. In this area, Chilean dolphins are mainly located in protected zones in fjords and channels [39,87]. Around 42°S latitude, the West Wind Drift reaches the coast and divides into the northward flowing Humboldt Current and the poleward Cape Horn Current, which passes around the continent through the Drake Passage, influencing both the east and west coasts of South America [91,92].

Geographic positions of biogeographic breaks are mainly determined by the coincidence in the limit of distribution of an unusual number of species [93]. Many species might cross these boundaries without seeming to “perceive” any discontinuity, while others exhibit strong genetic and even phylogeographic structure associated with the biogeographic breaks, depending on their ability to disperse and survive in different environmental conditions [93,94]. In many cases, the genetic structure pattern is generally considered as a consequence of limited gene flow associated with hydrography, coastal topography, temperature discontinuities and other factors [95,96]. In the Chilean dolphin, low migration rate values indicate restricted gene flow between populations, even when males and females were considered separately in order to detect sex-biased dispersal. As there is no evident physical barrier which contributes to the limited gene exchange between the North area and South area populations of the Chilean dolphin, the population differentiation may be a result of an integral (synergic) scenario of environmental factors and behaviors developed in different habitat types. Differences in oceanographic and topographic characteristics associated with geographic variation of prey items potentially contribute to the differentiation between these two populations. Chilean dolphin feeds on a wide variety of coastal prey, focusing on benthic and small pelagic schooling fish and squid [37]. The coastal fish fauna occurring from 30° to 42°S (central Chile) is a mixture of subAntarctic, subtropical, pan-oceanic and a few endemic fish species [97]. The dominant species among the small-sized fish consumers are anchovy, *Engraulis ringens*, and Pacific sardine, *Sardinops sagax*; large predators include the jack mackerel, *Trachurus murphyi*, hake, *Merluccius gayi*, and different cephalopod species [98]. The Chilean dolphin diet in this area seems to be composed mostly of sardine, *Strangomera bentincki*, anchovy and róbalo/Chilean rock cod, *Eleginops maclovinus* [34,37,38]. In contrast, over the continental shelf of the austral fjords region, the fish community is dominated by the demersal species as Patagonian grenadier, *Macruronus magellanicus* and southern hake, *Merluccius australis* [90]. Unfortunately, there is no published information about the diet of Chilean dolphin in the South area. In addition, a specialized behavior according to each particular area could also be an important factor that enforces the genetic structure in Chilean dolphin. However, behavioral studies in both areas should be conducted to evaluate hypothesis.

Habitat discontinuities associated with changes in oceanographic features, prey distribution and philopatric behavior have been previously proposed as influencing the spatial genetic structure of several delphinid species (e.g. [4,5,10,99,100]). In this context, the historical correlation of remote sensing environmental data (chlorophyll concentration, water turbidity and surface temperature) is relevant in the understanding of dolphin population structure (i.e., for the Franciscana dolphin *Pontoporia blainvillei* in the Western South Atlantic [100] and pilot whales in the North Atlantic [101]. Further studies on Chilean dolphin should incorporate the analysis of such oceanographic variables as well as diet composition differentiation to clarify the processes involved in shaping population differentiation.

### Conservation implications on the structuring of Chilean dolphin populations

Understanding population structure is crucial for informing management actions [102,103].The restricted migration rate and marked differentiation found between the North area and South area populations of the Chilean dolphin lead us to propose two different Management Units (MU). This operational description is usually used to guide short-term management issues, as it refers to current population structure and allele frequencies [14]. Thus, differentiated populations that are connected by low levels of gene flow are recognized as functionally independent. This management concept has been applied in different dolphin species as harbor porpoises in the North West Atlantic [57], bottlenose dolphin in Wider Caribbean [104] and the Hector’s dolphin in New Zealand [23].

In the case of Hector’s dolphin *C*. *hectori*, the North and South Island populations, exhibited a strong phylogeographic structure, evidencing the absence of gene flow between them [19,20]. Despite apparently not having a long-term evolutionary history, both populations fitted the ESU definition (Evolutionary Significant Units, [14]), and therefore, were elevated to subspecific status [18]. In this context, despite the biogeographical, genetic and habitat differences identified between the two *C*. *eutropia* North area and South area populations, our study does not provide a historical perspective that would allow if these populations could be categorized as an ESU and consequently, as potential subspecies. The use of additional molecular markers, such as mtDNA control region, would certainly bring important information about the long-term evolutionary history of *C*.*eutropia* that can be used to discuss their current taxonomy.

Our study provides valuable biological and ecological information for the Chilean dolphin, a species among the least known members of the family Delphinidae [105] and classified as Near Threatened by IUCN [41]. Considering the biogeographical, genetic and environmental differences between the two identified MUs of *C*. *eutropia*, it is necessary to recognize the current threats to which each of the Chilean dolphin populations are exposed, and design management strategies adjusted to each particular area if needed. This necessity is made more urgent by the apparently small effective size and the absence of any estimates for the census size of either population.

## Supporting Information

S1 Table Source of Chilean dolphin, *Cephalorhynchus eutropia*, samples and genetic sex identification per locality (DOCX)

S2 Table Microsatellite diversity of Chilean dolphin, *Cephalorhynchus eutropia*, for North Area and South Area (DOCX)
