# Supplementary material for: Microsatellite Markers Reveal Strong Genetic Structure in the Endemic Chilean Dolphin
Source: PLoS One. 2015 Apr 21;10(4):e0123956. doi: 10.1371/journal.pone.0123956 (PMC4405423; doi:10.1371/journal.pone.0123956)
Supplement: S1 Table — (DOCX) [file pone.0123956.s001.docx]

**S1 Table.** Source of Chilean dolphin*, Cephalorhynchus eutropia*, samples (successfully genotyped), genetic sex identification per locality.

| **Population** | **n** | **Skin swabbing** | **Biopsy darting** | **Beachcast** | **Males** | **Females** |
| --- | --- | --- | --- | --- | --- | --- |
| San Antonio | 1 |  |  | 1 |  | 1 |
| Constitución | 4 |  | 1 | 3 | 3 | 1 |
| Concepción | 9 |  | 7 | 2 | 8 | 1 |
| Maullín | 5 |  | 5 |  | 4 | 1 |
| Aysén | 10 | 8 | 2 |  | 1 | 9 |
| Bernardo O'Higgins | 7 |  | 7 |  | 5 | 2 |
| Puerto Natales | 12 |  | 12 |  | 10 | 2 |
| Punta Arenas | 5 |  | 4 | 1 | 1 | 4 |
| **Total** | **53** | **8** | **38** | **7** | **32** | **21** |
