# Supplementary material for: Microsatellite Markers Reveal Strong Genetic Structure in the Endemic Chilean Dolphin
Source: PLoS One. 2015 Apr 21;10(4):e0123956. doi: 10.1371/journal.pone.0123956 (PMC4405423; doi:10.1371/journal.pone.0123956)
Supplement: S2 Table — (DOCX) [file pone.0123956.s002.docx]

**S2 Table.** Microsatellite diversity of Chilean dolphin, *Cephalorhynchus eutropia*, for North Area and South Area populations per locus (* significant deviation from Hardy-Weinberg equilibrium, *p*<0.05). Number of sampled alleles (2n), Allelic richness (AR), observed heterozygosity (Ho) and expected heterozygosity (He).

|  | **North** | | | | | **South** | | | | |
| --- | --- | --- | --- | --- | --- | --- | --- | --- | --- | --- |
| **Locus** | **2n** | **# Alleles/ AR** | **Ho** | **He** | ***p*-value** | **2n** | **# Alleles/ AR** | **Ho** | **He** | ***p*-value** |
| Ev1 | 38 | 2 / 2.000 | 0.158 | 0.149 | 1 | 34 | 2 / 1.988 | 0.177 | 0.163 | 1.000 |
| Ev94 | 38 | 4 / 3.781 | 0.211 | 0.201 | 1 | 68 | 1 / 1.000 |  |  |  |
| Gt23 | 38 | 3 / 2.895 | 0.684 | 0.511 | 0.222 | 34 | 3 / 3.000 | 0.529 | 0.595 | 0.407 |
| Mk5 | 38 | 2 / 2.000 | 0.158 | 0.235 | 0.258 | 34 | 3 / 2.499 | 0.265 | 0.259 | 0.207 |
| Pph130 | 38 | 4 / 4.000 | 0.632 | 0.738 | 0.483 | 33 | 4 / 3.932 | 0.394 | 0.392 | 0.621 |
| Ev37 | 38 | 3 / 3.000 | 0.474 | 0.562 | 0.129 | 33 | 3 / 2.994 | 0.394 | 0.383 | 0.492 |
| Gt51 | 38 | 2 / 2.000 | 0.684 | 0.462 | 0.048* | 32 | 2 / 1.957 | 0.125 | 0.119 | 1.000 |
| Pph142 | 38 | 2 / 2.000 | 0.263 | 0.235 | 1 | 33 | 2 / 2.000 | 0.485 | 0.429 | 0.680 |
| Pph110 | 38 | 2 / 2.000 | 0.368 | 0.371 | 1 | 34 | 2 / 1.988 | 0.118 | 0.163 | 0.213 |
| Kwm12 | 38 | 5 / 4.886 | 0.684 | 0.653 | 0.909 | 32 | 6 / 5.390 | 0.594 | 0.650 | 0.074 |
| Pph137 | 38 | 3 / 2.895 | 0.421 | 0.351 | 1 | 33 | 4 / 3.515 | 0.788 | 0.666 | 0.207 |
| Gt211 | 36 | 4 / 3.944 | 0.556 | 0.690 | 0.366 | 20 | 4 / 4.000 | 0.550 | 0.642 | 0.417 |
| Mk6 | 38 | 2 / 2.000 | 0.632 | 0.512 | 0.378 | 28 | 4 / 3.605 | 0.500 | 0.460 | 0.731 |
| Sgui03 | 38 | 2 / 2.000 | 0.421 | 0.341 | 0.538 | 31 | 3 / 2.800 | 0.516 | 0.520 | 0.313 |
| Sgui06 | 34 | 6 / 6.000 | 0.941 | 0.749 | 0.402 | 32 | 6 / 5.307 | 0.688 | 0.652 | 0.474 |
| Sgui17 | 38 | 2 / 2.000 | 0.263 | 0.491 | 0.061 | 29 | 3 / 2.666 | 0.104 | 0.133 | 0.102 |
| Ev104 | 34 | 2 / 2.000 | 0.118 | 0.114 | 1 | 29 | 5 / 4.251 | 0.276 | 0.360 | 0.005* |
| Ev14 | 38 | 5 / 4.886 | 0.842 | 0.710 | 0.431 | 28 | 7 / 6.002 | 0.500 | 0.626 | 0.063 |
| Texvet5 | 38 | 3 / 3.000 | 0.474 | 0.605 | 0.310 | 28 | 8 / 6.596 | 0.571 | 0.600 | 0.005* |
| Sgui16 | 38 | 4 / 4.000 | 0.526 | 0.670 | 0.319 | 24 | 6 / 5.586 | 0.625 | 0.656 | 0.813 |
| Gt575 | 36 | 2/ 2.000 | 0.500 | 0.475 | 1 | 24 | 3 / 2.708 | 0.417 | 0.513 | 0.509 |
